# Homologous Recombination Is Stimulated by a Decrease in dUTPase in *Arabidopsis*

**DOI:** 10.1371/journal.pone.0018658

**Published:** 2011-04-26

**Authors:** Emeline Dubois, Dolores Córdoba-Cañero, Sophie Massot, Nicolas Siaud, Bertrand Gakière, Séverine Domenichini, Florence Guérard, Teresa Roldan-Arjona, Marie-Pascale Doutriaux

**Affiliations:** 1 Institut de Biologie des Plantes, Centre National de la Recherche Scientifique, Unité Mixte de Recherche 8618, Université Paris-Sud 11, Orsay, France; 2 Department of Genetics, University of Córdoba, Córdoba, Spain; 3 Plateforme métabolisme-métabolome, Institut de Biologie des Plantes, Université de Paris-Sud 11, Orsay, France; National Cancer Institute, United States of America

## Abstract

Deoxyuridine triphosphatase (dUTPase) enzyme is an essential enzyme that protects DNA against uracil incorporation. No organism can tolerate the absence of this activity. In this article, we show that dUTPase function is conserved between *E. coli* (*Escherichia coli*), yeast (*Saccharomyces cerevisiae*) and *Arabidopsis* (*Arabidopsis thaliana*) and that it is essential in *Arabidopsis* as in both micro-organisms. Using a RNA interference strategy, plant lines were generated with a diminished dUTPase activity as compared to the wild-type. These plants are sensitive to 5-fluoro-uracil. As an indication of DNA damage, inactivation of dUTPase results in the induction of *AtRAD51* and *AtPARP2*, which are involved in DNA repair. Nevertheless, RNAi/*DUT1* constructs are compatible with a *rad51* mutation. Using a TUNEL assay, DNA damage was observed in the RNAi/*DUT1* plants. Finally, plants carrying a homologous recombination (HR) exclusive substrate transformed with the RNAi/*DUT1* construct exhibit a seven times increase in homologous recombination events. Increased HR was only detected in the plants that were the most sensitive to 5-fluoro-uracils, thus establishing a link between uracil incorporation in the genomic DNA and HR. Our results show for the first time that genetic instability provoked by the presence of uracils in the DNA is poorly tolerated and that this base misincorporation globally stimulates HR in plants.

## Introduction

Deamination of cytosine (C) results in the formation of uracil (U) in DNA which will code for adenine in the following replication cycle, thus giving rise to a C to T (thymine) transition [1], [2]. DNA polymerase can also incorporate dUTP (deoxyuridine triphosphate) instead of dTTP (deoxythymidine triphosphate) [3]. To prevent such a misincorporation, a low dUTP to dTTP ratio must be maintained [4]. The enzyme deoxyuridine triphosphatase (dUTPase) hydrolyses dUTP to dUMP (deoxyuridine monophosphate) and pyrophosphate. dUMP can then be used as a substrate by thymidylate synthase to initiate the synthesis of dTTP. Through this reaction, the dUTPase performs two functions: keeping a low dUTP level in the cell and allowing dTTP synthesis. 5-fluoro-uracil (5FU) inhibits the thymidilate synthase thus decreasing dTTP synthesis, which leads to an increase in the dUTP/dTTP ratio which becomes more severe when the dUTPase is also depleted [5]. 5-FU can also incorporate into DNA, thus adding to the damage due to dTTP depletion, via its subsequent excision.

Uracils in DNA are repaired by the base excision repair (BER) pathway. They are first specifically recognized and excised by the uracil DNA glycosylase (UDG), leaving abasic sites [1] that can be processed through successive incision, resynthesis and ligation [6]. During the BER process, a single-strand break can be transformed into a double-strand break (DSB) if replication occurs at a nicked DNA site [7].

The gene coding for the dUTPase function is essential in *E. coli* (*Escherichia coli*) and yeast (*Saccharomyces cerevisiae*) and thus only leaky point mutations have been isolated for this gene [8], [9]. In *E. coli*, the *dut-1* mutant exhibits an increased homologous recombination (hyper-Rec) phenotype and is synthetically lethal with mutations in *RecA* or *RecBC*, two proteins that are central to the homologous recombination (HR) pathway [10], [11]. In yeast, a point mutation in the *DUT1* gene (*dut1-1*) triggers a spontaneous mutator phenotype [12] due to frequent erroneous BER repair. These observations show that uracils elimination in DNA is crucial, resulting in either increased HR or mutagenesis (or both).

HR is one of the mechanisms by which DNA DSBs can be repaired, even though it is not preponderant in plants [13]. It involves the search and recognition of an identical or homologous DNA sequence to repair the DSB. In eukaryotes, RAD51, the homolog of the *E. coli* RecA, is essential for these steps. In *C. elegans* (*Caenorhabditis elegans*), partial loss of dUTPase activity results in the accumulation of RAD51 foci in the chromatin suggesting that DSBs have been created and are being repaired by HR [14]. Decrease of dUTPase activity in *Trypanosoma* (*Trypanosoma brucei*), also results in the introduction of DSBs, as chromosome fragmentation is observed [15]. Taking together these results, we hypothesized that knocking-down dUTPase expression would lead to an increase in DSB formation and thus could influence HR levels in *Arabidopsis* (*Arabidopsis thaliana*).

In this article, we describe the consequences of a reduction in dUTPase activity in plants, in the continuity with our very preliminary published data [16]. We show that the *Arabidopsis DUT1* gene can functionally complement the *E. coli dut-1* mutant and yeast *dut1-1* mutant and that this gene is essential in *Arabidopsis*. In order to knock-down dUTPase expression, as no *dut1* mutant was available from the *Arabidopsis* collections of insertion lines, we produced RNAi/*DUT1* lines [16]. The RNAi/*DUT1* plants were effectively depleted in dUTPase activity, and showed sensitivity to 5FU. These plants exhibited *RAD51* and *PARP2* induction, and an increased frequency of HR events in their somatic cells, suggesting the presence of DNA damage and HR stimulation.

## Results

### *Arabidopsis DUT1* can complement a *dut-1* mutation in *E. coli* and *dut1-1* mutation in yeast

**Figure 1:**
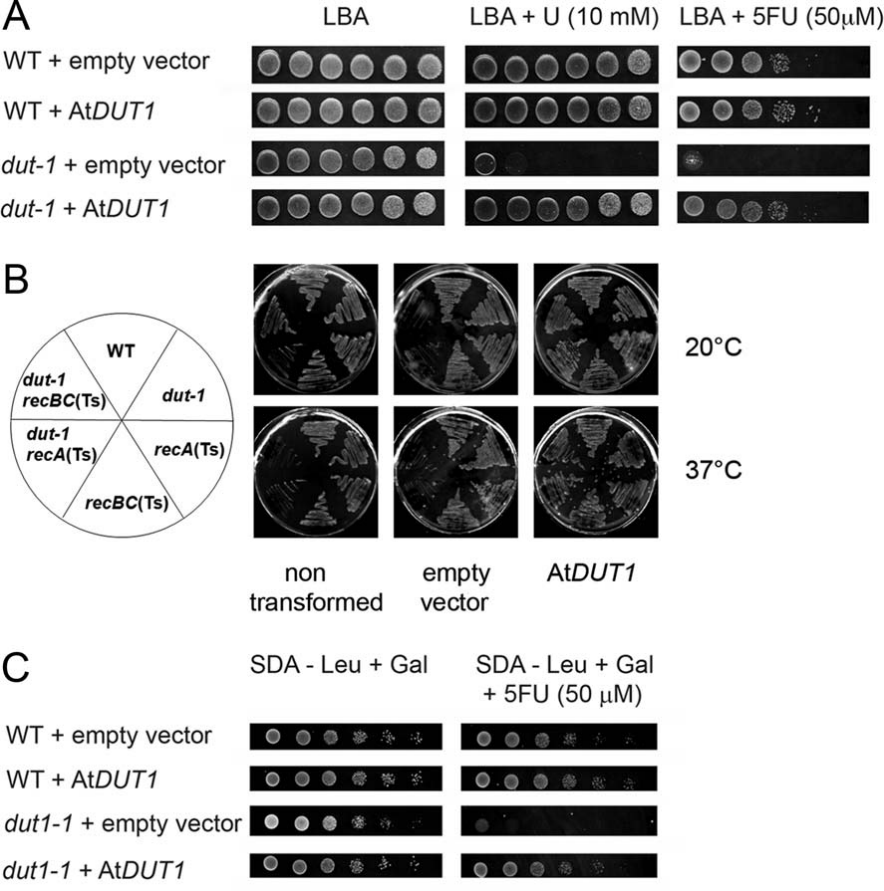
*Arabidopsis DUT1* can complement a *dut-1* mutation in *E. coli* and a *dut1-1* mutation in yeast. A – *E. coli* WT and *dut-1* strains transformed with an empty vector or a vector containing At*DUT1* were tested for their sensitivity to uracil (U) or 5-fluoro-uracil (5FU). B – *E. coli* WT, *recA*(Ts) or *recBC*(Ts) and *dut-1* or *dut-I recA*(Ts) and *dut-1 reBC*(Ts) strains were transformed with an empty vector or a vector containing At*DUT1* and grown at permissive (20°C) or non-permissive (37°C) temperature. C -Yeast WT and *dut1-1* strains transformed with an empty vector or a vector containing At*DUT1* were tested for their sensitivity to 5FU. Rapidly growing cultures were serially diluted 10-fold at each step and 15 µl of each were spotted in rows. The left most column is not diluted; the right most column is a 10^−5^ dilution.

The *dutΔ* (deletion) mutant is lethal in *E. coli*, but the *dut-1* partial inactivation (point mutation) mutant is viable and sensitive to the presence of uracil (10 mM) or an analog of uracil, 5FU (50 µM) [11], [17]. In order to examine the function of the *Arabidopsis DUT1* gene, we transformed the *E. coli dut-1* mutant with a plasmid expressing the At*DUT1* cDNA. The transformants were tested for their capacity to grow on uracil or 5FU containing media. Wild-type (WT) *E. coli* transformed with an empty vector remained sensitive to the presence of uracil or 5FU, whereas the growth of this same *dut-1* mutant on uracil or 5FU containing medium was restored to a similar level as the WT *E. coli* upon transformation with the At*DUT1* cDNA (Figure 1A). Similarly, *recA*(Ts) *dut-1* or *recBC*(Ts) *dut-1* double mutants are dead when grown at restrictive temperature (37°C). This lethality is rescued by At*DUT1* expression (Figure 1B).The *dut-1* phenotype caused by a mutation in the *E. coli dut* gene was thus rescued by expressing At*DUT1*.

The *dut1Δ* mutation is lethal in yeast, while a *dut1-1* leaky mutant (point mutation) is viable [12], but sensitive to the presence of 5FU (50 µM) (our data). Yeast transformed with either an empty control plasmid or a plasmid expressing the At*DUT1* cDNA were examined for their growth on 5FU-containing medium. The yeast *dut1-1* mutant transformed by an empty vector remained sensitive to 5FU, while yeast *dut1-1* transformed with the At*DUT1* cDNA grew as well as the WT on 5FU-containing media (Figure 1C). The 5FU sensitivity phenotype caused by a mutation in the Sc*DUT1* gene was thus rescued by the *Arabidopsis DUT1*. These results indicate that the dUTPase protein is functionally conserved between *E. coli*, *S. cerevisiae* and *Arabidopsis*.

### RNAi inactivation of *DUT1* expression *in planta* is lethal

**Figure 2:**
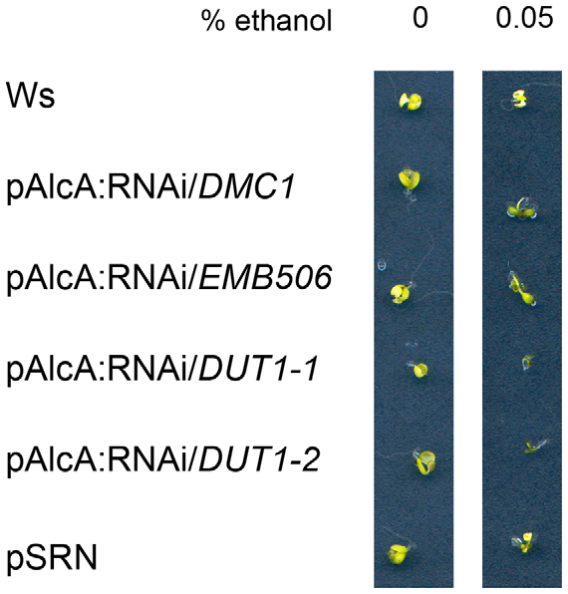
Effects of ethanol inducible expression on the viability of seedlings transformed by different RNAi constructs. Plants carrying the p35S:AlcR construct and a Nos terminator (PSRN) [19] were transformed with RNAi constructs under the pAlcA promoter in order to inactivate either *DMC1* (a meiotic gene), or *EMB506* (a gene which is embryo-lethal when mutated) or *DUT1* (two independent lines) expression. All the plants are of the same Ws ecotype as the WT or PSRN control seedlings.

Since no mutational insertion could be found in At*DUT1* in the *Arabidopsis* mutant collections, and since we suspected that such an insertion could be lethal, we depleted dUTPase in *Arabidopsis* using RNAi constructs. These constructs were set under the control of either the constitutive p35S promoter, or the meiotic p*DMC1* promoter [18], or the conditional pAlcA promoter [19]. Most of the primary transformants died prematurely, when the RNAi/*DUT1* construct was set under the p35S promoter [16], so we chose to study, among the surviving lines, two independent transformants named RNAi/*DUT1*-4 and RNAi/*DUT1*-5 which were the most sensitive to 5-FU. Both RNAi/*DUT1*-4 and RNAi/*DUT1*-5 lines also showed a floral phenotype as previously described in [16]. The lethality phenotype was specifically due to the RNAi/*DUT1* construct as it was not observed upon transformation with an empty vector (RNAi/0). Furthermore, it was partially alleviated upon the use of the p*DMC1* promoter [16]. Plants expressing AlcR (PSRN, p35S:AlcR tNOS), a transcription factor specific for pAlcA, which is activated under exposure to ethanol [19] were transformed with different RNAi constructs (*DUT1*, *DMC1* or *EMB506*) set under the pAlcA promoter and thus aimed at expressing the RNAi constructs under ethanol exposure. Only the lines containing the pAlcA:RNAi/*DUT1* construct died when grown on ethanol containing media (0.05%) (Figure 2). These data strongly suggest that a lack of dUTPase is lethal in *Arabidopsis*.

### *DUT1* expression and dUTPase activity are reduced in RNAi/*DUT1* lines

**Figure 3:**
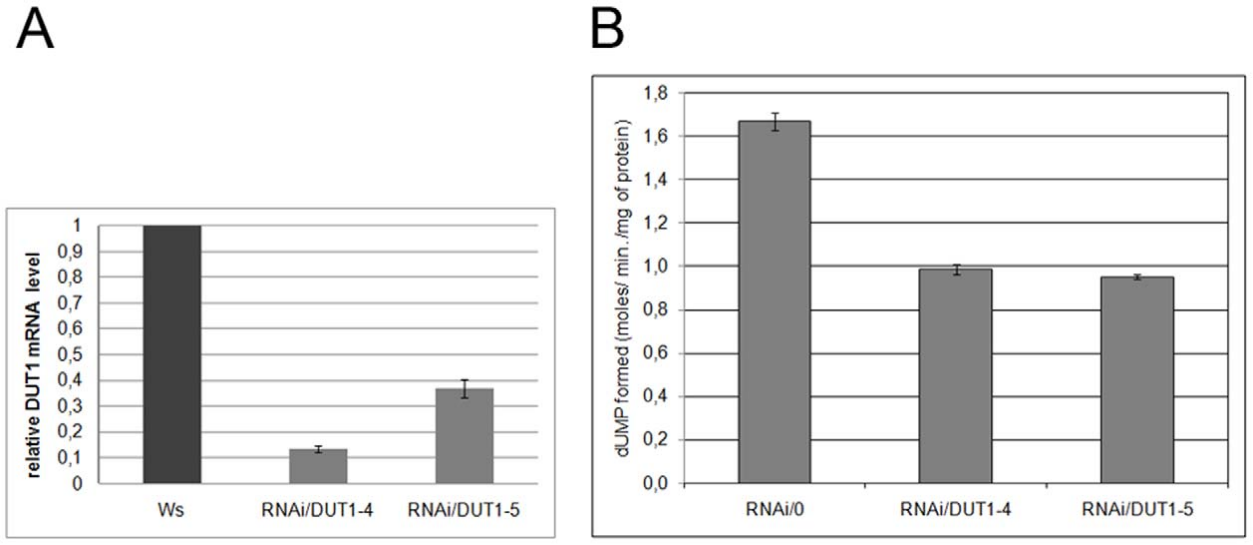
*DUT1* expression levels and dUTPase activities in RNAi/*DUT1* lines compared to control plants. A - *DUT1* expression level was analyzed by quantitative real time RT-PCR in young leaves of RNAi/*DUT1*-4 and -5 compared with WT plants (Ws ecotype). The relative mRNA normalized to *ACTIN* is reported. Error bars correspond to standard deviations. B - Level of dUTPase activity in RNAi/*DUT1-*4 and -5 plant lines compared to control plant (empty vector, RNAi/0). Activity was determined by measuring the dUMP produced by UPLC, after supplementation of plant extract with dUTP at 30°C for increasing lengths of time (three points in two independent experiments). Error bars correspond to standard deviations.

To assess the impact of the RNAi/*DUT1* construct on *DUT1* mRNA levels in the two independent RNAi/*DUT1*-4 and RNAi/*DUT1*-5 transgenic lines, chosen as 5FU sensitive, quantitative RT-PCR analyses were carried out. RNA extractions were performed on young leaves. The amount of *DUT1* mRNA detected in the RNAi/*DUT1*-4 and RNAi/*DUT1*-5 lines was decreased to 12% and 34%, respectively compared to WT plants (Figure 3A), while the steady-state level of the constitutive *ACTIN* gene was not affected.

To examine if this reduction in *DUT1* expression correlated with a decrease in dUTPase activity in the RNAi/*DUT1* lines, protein was extracted from the leaves of WT, RNAi/*DUT1*-4 and RNAi/*DUT1-5* plants and dUTPase activity was measured at 30°C using the crude extracts. The reaction was followed by measuring dUMP formation by UPLC (Ultra Performance Liquid Chromatography) after the addition of dUTP. The dUTPase activity in both RNAi/*DUT1* plants was found to be reduced by 40% of that measured in control plant (RNAi/0) extracts (Figure 3B). So, the RNAi/*DUT1* constructs were effective at decreasing At*DUT1* expression, and consequently the measurable *in planta* dUTPase activity.

### UNG activity is reduced in RNAi/*DUT1* lines

**Figure 4:**
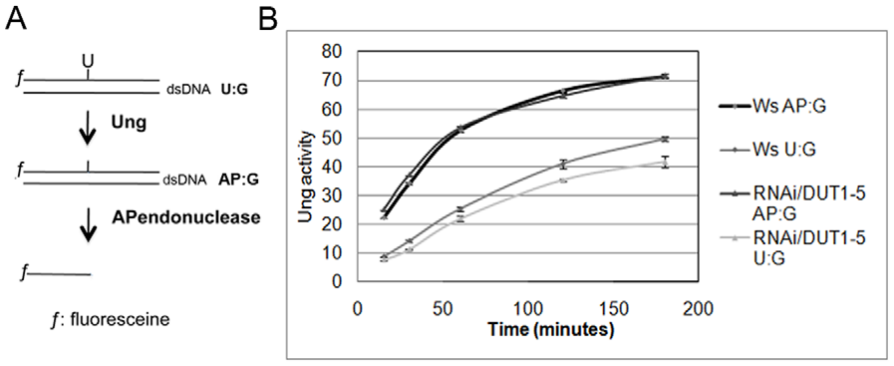
Kinetics of uracils excision reported to AP endonuclease activity. A - Schema showing the *in vitro* assay that allows to measure Ung activity. B - Protein extracts from Ws plants or RNAi/*DUT1*-5 plants were used to measure Ung followed by AP endonuclease activity (U∶G) as compared to AP endonuclease activity alone (AP∶G) at different time points. Error bars correspond to two independent experiments, done with two independent protein extracts.

Since in *Trypanosoma* [15], abnormally high levels of uracil in DNA induce a change in BER activities, we measured *in vitro* uracil excision (U∶G) and AP site (AP∶G) repair activities in protein extracts comparing WT to RNAi/*DUT1* plants, as described in [20], [21] (Figure 4A). We compared the repair kinetics of uracil versus apurinic site with the same amounts of protein extracted from either WT or RNAi/*DUT1* seedlings. It should be noted that to measure UNG activity, a substrate that contains a uracil (U∶G) is added to the extract, and this substrate must first undergo uracil excision, and then apurinic site endonucleotytic cleavage for the UNG activity to be detected. Therefore, UNG activity is the sum of both uracil excision and apurinic site incision. Unexpectedly, we observed that the level of UNG activity was diminished by 30 to 40% in the RNAi/*DUT1* plant protein extracts compared to the WT (Figure 4B). We therefore hypothesized that UNG activity was down-regulated in RNAi/*DUT1* plants. The AP endonucleolysis itself was not affected in the RNAi/*DUT1* seedlings compared to the WT. This down-regulation of the UNG activity could prevent too many uracil excisions to occur at the same time since this might be toxic for the plant DNA.

### DNA fragmentation is observed in RNAi/*DUT1* lines

**Figure 5:**
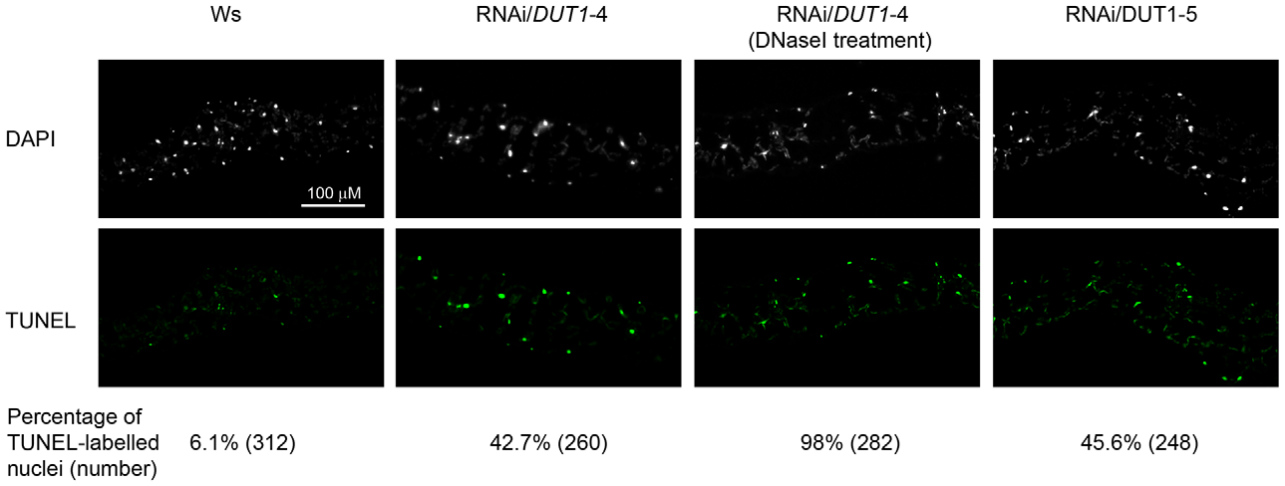
TUNEL analysis of genomic fragmentation in WT and RNAi/*DUT1* lines. WT (Ws) and RNAi/*DUT1* leaf tissues were subjected to TUNEL labelling and observed by fluorescence microscopy. Numbers in parenthesis indicate the number of nuclei that were observed. Nuclear DNA was stained with DAPI.

To investigate whether the decrease in dUTPase activity resulted in increased breaks in the genomic DNA, as in *Trypanosoma* [15], we performed a TUNEL assay. This procedure is used to label DNA strand breaks. We exposed transversal leaf sections to terminal deoxynucleotidyltransferase enzyme-mediated dUTP end labelling (TUNEL). In such an experiment, nuclei are labelled if strand breaks are present. Only DNA breaks that have become labelled with the modified nucleotides that are extraneously brought due to the kit system can be observed. As a control, RNAi/*DUT1*-4 leaves were treated with DNAse I and this led to 98% breaks in their DNA. Leaves from WT plants showed 6.1% labelled nuclei. In RNAi/*DUT1-4* and RNAi/*DUT1-5* lines, this percentage reached 42.7% to 45.6%, respectively (Figure 5). The observation that DNA staining was slightly more efficient in lines RNAi/*DUT1-5* versus RNAi/*DUT1-4* may also indicate that dUTPase activity *per se* does not really influence the assay. The RNAi/*DUT1* plants thus show increased DNA fragmentation which is compatible with uracil excision and apurinic site incision and thus the formation of DNA breaks but this was not associated with apoptosis as the cells looked intact (Figure 5). Furthermore, this was not the result of apoptotic DNA breakage as DNA extracted from the same leaves showed the expected high molecular weight migration (data not shown).

### *RAD51* or *PARP2* expression are induced in RNAi/*DUT1* plants

**Figure 6:**
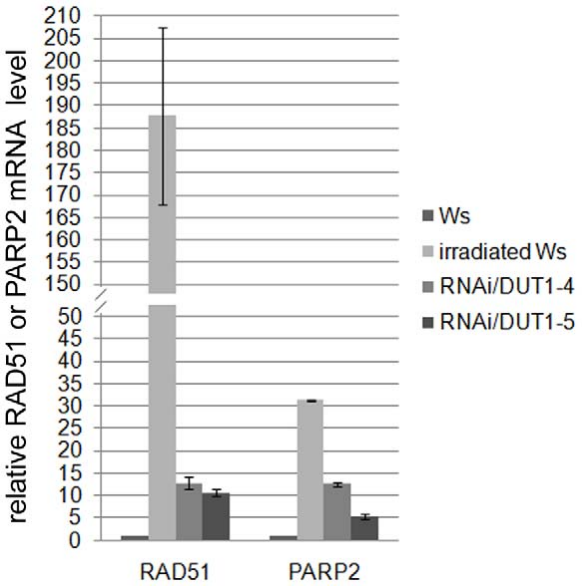
Expression level of *RAD51* and *PARP2* in RNAi/*DUT1* lines compared to control plants (WT, irradiated or not). *RAD51* and *PARP2* expression in young leaves of RNAi/*DUT1*-4 and -5 was compared to WT plants (Ws), irradiated or not (Gamma rays at a 10 Krad dose). The relative mRNA normalized to *ACTIN* is reported. Error bars correspond to standard deviations.

In plants, *RAD51* is strongly induced following gamma irradiation which produces DNA DSBs [22], [23]. To assess the impact on *RAD51* expression in the RNAi/*DUT1-4* and RNAi/*DUT1-5* lines, quantitative real-time-PCR analyses were conducted. RNA extraction was performed from young leaves. As a positive control of *RAD51* induction, we analyzed RNA in young leaves one hour after gamma irradiation (100 Gray) of WT plants. Compared to non-irradiated plants, *RAD51* was induced 230 times in irradiated plants and 13- to 11- fold, respectively in the RNAi/*DUT1-4* and RNAi/*DUT1-5* plants, compared to WT (Figure 6). *RAD51* induction in both RNAi/*DUT1-4* and RNAi/*DUT1-5* lines was previously observed by Northern blot analysis [16] but this method did not allow for a proper quantification. To confirm that this could be generalized to other gamma-induced genes, we tested *PARP2* which is also induced following gamma irradiation of *Arabidopsis* [23]. The results were similar to *RAD51* in that *PARP2* expression was increased 31 fold in WT irradiated plants versus non-irradiated WT plants and 12- and 5- fold in RNAi/*DUT1-4* and RNAi/*DUT1-5* plants respectively, compared to WT plants (Figure 6). This indicates that DNA damage occurs in RNAi/*DUT1* plant lines, albeit to a lesser extent than after a 100 Gray gamma irradiation, but it must be continuous as the p35S promoter is constitutive and thus active all along the plant development.

### RNAi/*DUT1* is compatible with a *rad51* ^−/−^ mutation in *Arabidopsis*

**Figure 7:**
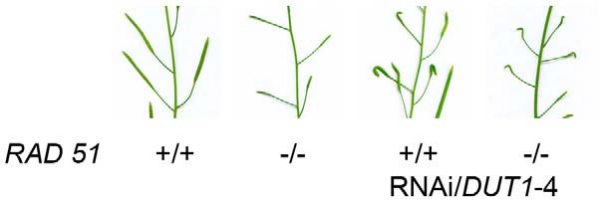
RNAi/*DUT1*-4 siliques in a WT versus *rad51* ^−/−^ background. Siliques were found to have the same “unfolded” floral phenotype appearance in the RNA/*DUT1*-4 *rad51* ^−/−^ mutant plants as in the WT plants, while also being shorter due to the sterility of the *rad51* ^−/−^ mutation.

In *E. coli*, *dut-1* is synthetically lethal with a *recA* mutation [11], suggesting that DSBs resulting from a decrease in dUTPase activity are repaired by HR. In eukaryotes, the RecA homolog is RAD51. In *Arabidopsis*, the *rad51^−/−^* mutant is viable, but sterile [24]. Since we observed that *AtRAD51* was induced in the RNAi/*DUT1* plants, we hypothesized that this gene would be necessary to repair lesions caused by a reduced dUTPase activity, similarly to the lethality of the *recA dut-1* double mutants in *E. coli*. In order to know if the RNAi/*DUT1* construct is compatible with the *rad51* ^−/−^ mutation in *Arabidopsis*, we crossed a RNAi/*DUT1*-4 plant with a *Rad51* ^+/−^ plant. We obtained ¼ of *rad51^−/−^* RNAi/*DUT1* in the F2 progeny, which corresponds to the expected percentage of segregating homozygous mutant plants. The *rad51^−/−^* RNAi/*DUT1* plants are as sterile as the *rad51^−/−^* plants and exhibit the same conspicuous defect in floral growth as the RNAi/*DUT1*-4 plants (Figure 7) [16]. This observation suggests that repair of DSBs resulting from a decreased dUTPase activity is not entirely dependent on HR or on *AtRAD51* as suggested from its induction. However, it has already been proposed that NHEJ is the main DSB repair route in plants [13]. Our data confirm that HR occurs but that it is not the only pathway for DSB repair in the RNAi/*DUT1* plants, although *RAD51* was found to be induced in these same plants.

### Somatic homologous recombination

**Figure 8:**
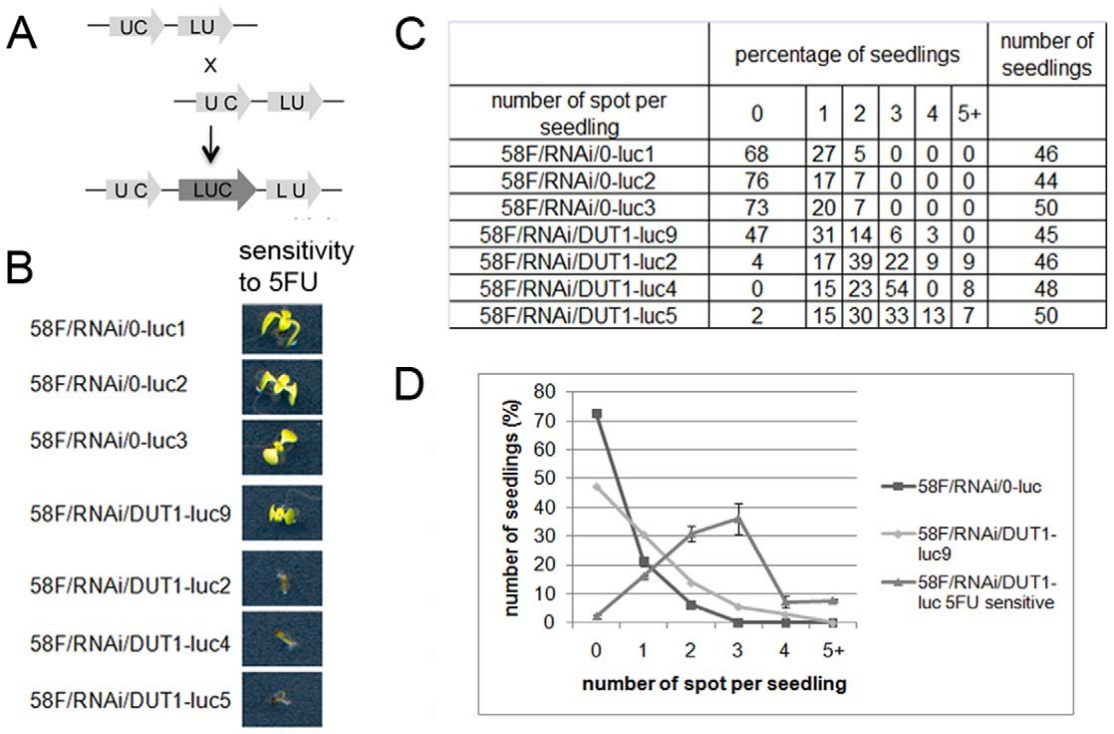
Frequency of homologous recombination events in relation to the 5FU sensitivity of RNAi/*DUT1* lines and control plants. A – Schema showing the luciferase *in planta* substrate for recombination that allows to measure HR. B - We compared the 5FU sensitivity (50 µM) of three independent transformants and then we gathered the empty vector control together (58F/RNAi/0-luc), the 5FU sensitive RNAi/*DUT1* lines together (58F/RNAi/*DUT1*-luc-5FUsensitive) and the 5FU resistant RNAi/*DUT1* line alone (58F/RNAi/*DUT1*-luc9), with the aim to represent the trend lines in the homologous recombination assay. C – Table showing the percentage of seedling we counted with different number of luciferase foci. D – Three independent control transformants were used (58F/0-luc1, -luc2 and -luc3) and four independent RNAi/*DUT1* transformants, 58F/RNAi/*DUT1*-luc2, -luc4, -luc5 and -luc9 to assess somatic recombination in line 58F.

If inactivation of *DUT1* in *Arabidopsis* leads to the formation of DNA breaks, and if this causes *RAD51* induction, these breaks could be repaired by HR. To determine the frequency of HR in RNAi/*DUT1* plants, plants carrying an artificial construct allowing the reconstitution of a functional luciferase reporter gene upon repair by HR only was used (line 58F) [25] (Figure 8A). The RNAi/*DUT1* construct was introduced into plant line 58F by transformation. The sensitivity to 5FU was assessed in selected plants in order to establish the RNAi silencing efficiency of the *DUT1* gene in these new transformants (Figure 8B). Three plant lines were sensitive to 5FU (58F/RNAi/*DUT1*-luc2, -luc4 and -luc5), whereas one (58F/RNAi/*DUT1*-luc9) was as 5FU resistant as plants transformed with the empty vector, and was thus considered as not depleted in dUTPase activity. As a control, we selected three independent lines called 58F/RNAi/0-luc1, -luc2 and -luc3, which were transformed with an empty RNAi vector. The level of HR was monitored as the frequency of luciferase foci per seedling (Figure 8C and 8D). In the majority of the 58F/RNAi/0 and in the 58F/RNAi/*DUT1*-luc9 plants, recombination events were not detected. However, in the 58F/RNAi/*DUT1*-luc2, -luc4 and -luc5 lines, we detected more than two recombination events per seedling (Figure 8C and 8D). Another way to measure these HR events is to report the frequency of foci per plants. The three lines 58F/RNAi/0 showed 0.34+/−0.03 foci/plantlet, while the three lines 58F/RNAi/*DUT1* showed 2.51+/−0.11 foci/plantlet. The frequency of HR events in the RNAi/*DUT1* plants was thus increased by a factor of 7.4 as compared to plants transformed with an empty vector. So a decrease in dUTPase activity increases the occurrence of recombination events in *Arabidopsis*. Such a result has never been obtained previously by directly acting on an *Arabidopsis* function.

## Discussion

Our data clearly show that dUTPase activity is essential in *Arabidopsis*, as previously observed in most of the organisms where it has been studied. Participation in dTTP synthesis, protection against uracil incorporation into DNA, repair of these uracils once in the DNA are at the heart of this property. No cell can tolerate the high level incorporation of uracils, whether it is due to the presence of uracils in DNA itself, or to their repair, once incorporated. Thus, dUTPase activity is extremely conserved, as seen by the fact that the *Arabidopsis* cDNA can complement *E. coli* as well as yeast dUTPase mutants. This conservation is also attested by the level of conservation between the respective proteins: the AtDut1 protein has 30.6% and 44.9% identity with the EcDut1 and ScDut1 proteins respectively. Only point mutants can be isolated in these microorganisms, and they are thus partial, while deletions are found to be lethal. It is rare that a gene from *Arabidopsis* can complement a defect in both bacteria and yeast. For instance, *RAD51* does not complement a *recA* mutation in *E. coli* or a *rad51* mutation in yeast (our personal data). The effectiveness of our dUTPase complementation also suggests that this protein has no critical partners, the independent evolution of which would have precluded it to be functional in different backgrounds.

Since no insertional mutants were available in *Arabidopsis*, we obtained, *via* a RNAi strategy, plants in which dUTPase activity was partially decreased. This decrease was attested at the transcription and the enzyme activity levels. It led to reproducible phenotypes that corroborate the fact that a lack of dUTPase is deleterious to cells in plants as previously shown in *E. coli* and yeast. It should be noted that such a conservation of phenotype between *E. coli* and *Arabidopsis* is not always observed. These plants were extremely sensitive to 5FU, grew poorly, died when the RNAi construct was induced, showed DNA breaks as indicated by the “TUNEL assay”, and induced genes specific to the DNA-damage response (*PARP2*, *RAD51*) in response to these DNA breaks.

However, crossing our RNAi/*DUT1* lines with an *Arabidopsis rad51* mutant showed that they were compatible. This is not always the case in micro-organisms such as *E. coli* where the *dut1-1* mutation becomes lethal when accompanied by either *recA*(Ts) or *recB*(Ts) mutations when grown at non-permissive temperature [11]. This clearly suggests that most DNA breaks in *Arabidopsis* are not repaired *via* the HR pathway, as previously discussed [13], while they are in *E. coli*. This appears to be an important difference between prokaryotes and eukaryotes since the absence of a functional homologous recombination repair pathway is most detrimental to *E. coli* while it is well-tolerated in higher eukaryotes such as *Arabidopsis*.

Finally, our data show that HR is probably increased in the RNAi/*DUT1* plants provided, and only when, the RNAi is efficient at decreasing the dUTPase activity (which is ascertained by the 5FU sensitivity of the transgenic RNAi/*DUT1* plants). This occurs consequently to a modification of the DNA itself without the introduction of any recombinase. A comparable result was observed when similar reporter lines were exposed to genotoxic stresses such as methyl-methone-sulfonate or bleomycin that are known to induce double strand breaks [25]. The inner DNA metabolism was changed in the RNAi/*DUT1* plant cells, DSBs were formed as an effect of the reduced dUTPase activity. HR was increased seven-fold in the RNAi/*DUT1* plants even though it was quantified with a construct that only presents 1146 bp of homology. The chances of having a DSB occurring in this region are lower than when an I-SceI site is introduced and used as an initiator for HR once cut, which increased HR by a factor of up to two orders of magnitude in tobacco cells [13]. However, one might expect that by extending the homology, somatic HR *via* a reduction in dUTPase activity might become very efficient. Therefore, knocking down *DUT1* may be a good tool to obtain gene targeting in plants.

## Methods

### Plasmid construction

The *DUT1* cDNA was cloned into pSE380 (Promega) for expression in *E. coli* (*Escherichia coli*) and into *p415GAL1* for expression into yeast (*Saccharomyces cerevisiae*) [26]. Vectors for p35S (constitutive) or p*DMC1* (meiotic) expression in plants are described in Siaud *et al.* [16], [18]. The pAlcA T-DNA vector for conditional expression in plants was made by introducing the pAlcA promoter [19] into the vector pPF111. *DUT1* is named K68 in Siaud *et al.* (2010) [16]. RNAi constructs were as in Siaud *et al.* [16], [18] and set under the control of the p35S, p*DMC1* or pAlcA promoters in the different experiments.

### Strains

*E. coli dut-1* mutants were provided by A. Kuzminov [11]: wild-type (WT), AB1157 (F^−^ l^−^ rac^−^ *thi-1 hisG4* Δ*(gpt-proA)62 argE3 thr-1 leuB6 kdgK51 rfbD1 araC14 lacY1 galK2 xylA5 mtl-1 tsx-33 supE44(glnV44) rpsL31(strR)*; *dut-1* (AK105 *dut-1 zic-4901*::Tn*10*: AB1157×P1 PK4001); the WT, *recA*(Ts) and *recBC* (Ts) ± the *dut1-1* mutation were as in [11] as well.

Yeast *dut1-1* mutant was provided by S. Boiteux [12]: wild-type (WT) corresponds to FF18733 (*MAT* ***a*** *leu2-3,112 trp1-289 his7-2 ura3-52 lys1-1*) and *dut1-1* to BG217 (FF18734 with *dut1-1*, *ade2Δ*).

### Complementation of *E. coli dut-1* or yeast *dut1-1* mutants

Transformed *E. coli* were selected as ampicillin resistant. Depending on the assay, plates were examined in the presence or absence of uracil (10 mM) or 5FU (50 µM) at 37°C. When examining thermosensitive mutations, plates were incubated for 24–36 h at the required temperature (20°C or 37°C). Transformed yeasts were selected on SD-Leucine, their promoter was induced with galactose and they were examined in the presence or absence of 5FU (50 µM). The plates were incubated for 48 h at 30°C.

### Plant Growth Conditions

*Arabidopsis thaliana* plants (ecotype Columbia, Col-0 or Wassilewskija, Ws) were grown in the greenhouse with a photoperiod of 16 h light and 8 h dark at 23°C or *in vitro* on MS 0.5 (Duchefa, NL) with or without 5FU (50 µM). The pAlcA promoter constructs were examined *in vitro* with or without ethanol at 0.05%. Plants carrying p35S::AlcR construction with a Nos terminator (pSRN) are described in [19]. Plants with the luciferase (58F) were used to measure recombination events, are described in [25]. The *Arabidopsis rad51* mutant is described in [24]. Basta resistant transformants were selected under greenhouse conditions.

### Quantitative real-time-PCR analysis

Total RNA from young leaves were isolated with the Nucleospin kit (Macherey-Nagel). Reverse transcription was performed using the 〈〈SuperScript™ First-Strand Synthesis System for RT-PCR〉〉 kit (Invitrogen) according to the manufacturer instructions. Real-time amplifications were performed in a LightCycler® 480 Real-Time PCR System (Roche) with LightCycler® 480 SYBR Green I Master gene using specific primers: *DUT1* (*DUT1* fwd: tcgattcccaatttcaacaa; *DUT1* rev: atcgtatccagcggagagtg); *ACTIN* (*ACT* fwd: ggtaacattgtgctcagtggtgg; *ACT* rev: aacgaccttaatcttcatgctgc); *RAD51* (*RAD51* fwd: cttagggatgctggtctctgtac; *RAD51* rev: gtcaaccttggcatcactaattc); *PARP2* (*PARP2* fwd: tcaaatccagtaatgggacaga; *PARP2* rev: tccgactctaggacttgtagga) and 1 µL of cDNA in a total volume of 25 µl. Each experiment, from RNA extraction to cDNA synthesis, was performed twice independently, and quantitative RT-PCR was performed using triplicate samples. The relative level of mRNA reported in the figures was normalized using the *ACTIN* mRNA level.

### Protein extracts for dUTPase activity and dUTPase activity

Total soluble proteins were extracted from 0.3 g of leaves from two month-old plants. Leaves were collected and crushed in the presence of liquid nitrogen and then 1 ml of extraction buffer was added: 100 mM Tris-HCl (pH 7.5), 20 mM MgCl2, 5 mM DTT and 1 µl of protease inhibitor (Protease inhibitor cocktail for plants, Sigma-Aldrich). The suspension was centrifuged at 14 000 g for 10 min at 4°C and supernatants were desalted on a NAP-5 column (GE-Healthcare) and eluted with extraction buffer. Protein concentrations were measured according to Bradford et al (1976) [27]. The dUTPase activity assay was performed in 100 mM Tris-HCl (pH 7.5), 20 mM MgCl_2_, 100 µM dUTP (Sigma-Aldrich) at 30°C. The reaction was initiated by adding protein extract and aliquots were taken at different times. The reaction was stopped by heating to 100°C. After centrifugation, the supernatant was subjected to reverse-phase ultra performance chromatography (AcquityTM UPLC, Waters) using a UPLC HSS T3 1.8 µm (100 mm•2.1 mm i.d.) column (Waters). dUMP formed in the course of the reaction was eluted using a linear gradient of 5% methanol in 15 mM Triethylamine pH 7.9 (0.6 mL.min-1) and detected at 260 nm using a diode array detector (Waters 2696 PDA detector). Peak areas were calculated using the Empower software (Waters), using commercial dUMP as a standard (Sigma). To ensure that steady-state conditions were applied, dUMP was measured at three time points. Product formation was linear for at least 45 min.

### Protein extracts for Ung activity

0.5 g of leaves were crushed in the presence of liquid nitrogen and 1.5 mL of extraction buffer was added (25 mM Hepes KOH at pH 7.8, 100 mM KCl, 5 mM MgCl_2_, 250 mM Saccharose, 10% Glycerol, 1 mM DTT) with 16 µL of protease inhibitor cocktail (Sigma-Aldrich). After one hour in ice and a centrifugation, supernatant was filtered and dialyzed in dialysis buffer (25 mM Hepes KOH at pH 7.8, 100 mM KCl, 17% glycerol, 2 mM DTT). Protein concentrations in the extracts were measured according to Bradford and collaborators (Bradford, 1976) [27].

### DNA substrates for Ung activity

Oligonucleotides used as DNA substrates for this assay are described in [20], [21]. U∶G duplex correspond to Fl-UGF, CGR-G and AP∶G duplex correspond to Fl-APGF and CGR-G sequences. Oligonucleotides were synthesized by Operon and were purified by PAGE before use. Double-stranded DNA substrates were prepared by mixing a 5 µM solution of a 5′-fluorescein-labelled oligonucleotide (upper-strand) with a 10 µM solution of an unlabelled oligomer (lower-strand), heating to 95°C for 5 minutes and slowly cooling to room temperature.

### Ung activity

Double-stranded oligodeoxynucleotides (2 pmol) were incubated at 30°C for 3 hours in a reaction mixture containing, 2.5 mM Hepes KOH at pH 7.8, 10 mM KCl, 2.5% Glycerol, 1 mM DTT, 0.2 mM EDTA and 10 µg of protein extract in a total volume of 50 µl. Reactions were stopped by adding EDTA to 20 mM, sodium dodecyl sulfate to 0.6%, and proteinase K to 200 µg/ml, and the mixtures were incubated for 30 min at 37°C. DNA was extracted with phenol∶chloroform∶isoamyl alcohol (25∶24∶1) and ethanol precipitated at −20°C in the presence of 0.3 mM NaCl and 16 µg/ml glycogen. Samples were resuspended in 10 µl of 90% formamide, and heated at 95°C for 5 min. Reaction products were separated in a 12% denaturing polyacrylamide gel containing 7 M urea. Fluorescein-labelled DNA was visualized using the blue fluorescence mode of the FLA-5100 imager and analysed using Multigauge software (Fujifilm).

### TUNEL assay

The TUNEL assay was conducted as in [28] and following the manufacturer's instructions (Roche Applied Science).

### Luciferase activity

The detection of bioluminescence was performed on two-week-old T2 generation seedlings of independent lines. Seedlings were sprayed with a solution of Luciferin (1 mM Luciferin (Biosynth AG), 0.05% Triton X-100) and visualized with a LCD camera used with the Photolite software. The number of spots per plant was then measured. Spots indicate the presence of a reconstituted active luciferase gene and hence occurrence of recombination events.
